# Master Regulators of Epithelial-Mesenchymal Transition and WNT Signaling Pathways in Juvenile Nasopharyngeal Angiofibromas

**DOI:** 10.3390/biomedicines9091258

**Published:** 2021-09-18

**Authors:** Naiade Calanca, Sara Martoreli Silveira Binato, Sabrina Daniela da Silva, Helena Paula Brentani, Luiz Ubirajara Sennes, Clóvis Antonio Lopes Pinto, Maria Aparecida Custódio Domingues, Carlos Eduardo Fonseca-Alves, Claudia Aparecida Rainho, Silvia Regina Rogatto

**Affiliations:** 1 Department of Chemical and Biological Sciences, Institute of Biosciences, São Paulo State University (UNESP), Botucatu 18618-689, Brazil; naiade.calanca@unesp.br (N.C.); claudia.rainho@unesp.br (C.A.R.); 2 International Research Center, CIPE, A.C. Camargo Cancer Center, São Paulo 01508-010, Brazil; s.m.binato@uni9.pro.br; 3 Department of Otolaryngology—Head and Neck Surgery, McGill University, Sir Mortimer B. Davis-Jewish General Hospital, Montreal, QC H3A 1A1, Canada; sabrina.wursba@mcgill.ca; 4 Department of Psychiatry, LIM23 (FMUSP), University of São Paulo (USP), São Paulo 05403-010, Brazil; helena.brentani@fm.usp.br; 5 Department of Otorhinolaryngology, LIM23 (FMUSP), University of São Paulo (USP), São Paulo 05403-010, Brazil; lsennes@usp.br; 6 Department of Pathology, A.C. Camargo Cancer Center, São Paulo 01509-010, Brazil; calopes@accamargo.org.br; 7 Department of Pathology, Faculty of Medicine, São Paulo State University (UNESP), Botucatu 18618-687, Brazil; maria.domingues@unesp.br; 8 Institute of Health Sciences, Paulista University—UNIP, Bauru 17048-290, Brazil; carlos.e.alves@unesp.br; 9 Department of Veterinary Surgery and Anesthesiology, São Paulo State University (UNESP), Botucatu 18618-687, Brazil; 10 Department of Clinical Genetics, University Hospital of Southern Denmark, 7100 Vejle, Denmark; 11 Institute of Regional Health Research, University of Southern Denmark, 5000 Odense, Denmark

**Keywords:** juvenile nasopharyngeal angiofibroma, gene expression profile, WNT pathway, tumor signaling pathways, tumor microenvironment, epithelial–mesenchymal transition

## Abstract

Juvenile nasopharyngeal angiofibroma (JNA) is a rare fibrovascular benign tumor showing an invasive growth pattern and affecting mainly male adolescents. We investigated the role of epithelial–mesenchymal transition (EMT) and WNT signaling pathways in JNA. Gene expression profiles using nine JNA paired with four inferior nasal turbinate samples were interrogated using a customized 2.3K microarray platform containing genes mainly involved in EMT and WNT/PI3K pathways. The expression of selected genes (*BCL2*, *CAV1*, *CD74*, *COL4A2*, *FZD7*, *ING1*, *LAMB1*, and *RAC2*) and proteins (BCL2, CAV1, CD74, FZD7, RAF1, WNT5A, and WNT5B) was investigated by RT-qPCR (28 cases) and immunohistochemistry (40 cases), respectively. Among 104 differentially expressed genes, we found a significantly increased expression of *COL4A2* and *LAMB1* and a decreased expression of *BCL2* and *RAC2* by RT-qPCR. The immunohistochemistry analysis revealed a low expression of BCL2 and a negative to moderate expression of FZD7 in most samples, while increased CAV1 and RAF1 expression were detected. Moderate to strong CD74 protein expression was observed in endothelial and inflammatory cells. A significant number of JNAs (78%) presented reduced WNT5A and increased WNT5B expression. Overall, the transcript and protein profile indicated the involvement of EMT and WNT pathways in JNA. These candidates are promising druggable targets for treating JNA.

## 1. Introduction

Juvenile nasopharyngeal angiofibroma (JNA) is a benign mesenchymal neoplasm characterized by a high vessel density that occurs almost exclusively in male adolescents and young adults. Imaging analyses have demonstrated that the tumor arises from the choana region and nasopharynx [1]. Despite its benign status, JNA presents a locally infiltrative growth pattern that often displays skull base invasion at diagnosis and less commonly spreads through the orbital fissure, resulting in an intracranial extension [2]. JNAs represent less than 0.5% of all head and neck tumors, and the estimated recurrence rate ranges from 5% to 25% according to the World Health Organization (WHO) classification of head and neck tumors [3]. Whereas the etiopathogenesis of these tumors remains elusive, there is evidence suggesting the potential influence of sex hormones and genetic factors, including familial adenomatous polyposis [4,5]. In a limited number of cases, the upregulation of pro-angiogenic growth factors and infection with human papillomavirus (HPV) and Merkel cell polyomavirus (MCPyV) have also been described [5,6,7].

Since JNAs are highly vascularized, the search for deregulated pro-angiogenic growth factors has drawn particular attention. The overexpression of vascular endothelial growth factor A (VEGFA) has been consistently reported in several studies [8,9,10,11]. Although some studies detected VEGF primarily in endothelial cells, others reported its presence in stromal and endothelial cells, or even predominantly in stromal cells [12]. Increased expression levels of fibroblast growth factor (FGF) and fibroblast growth factor receptor (FGFR) have also been described in JNAs [8,10,11,13]. The FGF/FGFR signaling axis modulates mitosis and cell survival, differentiation, and migration [14]. In a previous study, our group reported the overexpression of *FGF18* in endothelial and stromal components of JNAs compared to normal cells [13]. *FGF18* is a downstream target of the activated WNT pathway, whose aberrant signaling has been implicated in tumorigenesis, metastasis, and other human diseases [15,16,17].

In the last decade, a few studies described the involvement of specific genes and signaling pathways in JNAs. The amplification and overexpression of *CTCFL* (CCCTC-binding factor like) and *TSHZ1* (teashirt zinc finger homeobox 1) were reported [18]. In another study, copy number alteration and gene and protein expression analyses were performed to compare two JNAs presenting low- and high-stage at diagnosis [19]. The gene expression analysis revealed 1245 differentially expressed genes (DEGs). In this comparison, only three genomic imbalances presented correspondent altered gene expression. The authors described that the WNT-activated receptor activity was enriched in high-stage tumors. RNA sequencing analysis was performed comparing primary fibroblasts from two JNA explants and tumor-free tonsil tissues, revealing 1088 DEGs [10]. Based on this result, an ingenuity pathway analysis (IPA) was carried out, predicting the activation of VEGF and FGFR signaling.

Herein, we evaluated the gene expression profile in nine JNAs using a customized microarray platform. A subset of selected genes and proteins was investigated in a series of cases that expanded the one used for the array analysis. The results underscored molecules that might be explored for targeted JNA therapy.

## 2. Materials and Methods

### 2.1. Patients

Twenty-eight JNA fresh frozen samples and eight inferior nasal turbinate (INT) specimens from male individuals were obtained from the Department of Otorhinolaryngology of the University of São Paulo (São Paulo, SP, Brazil). Imaging evidence suggests that JNA originates from the region of the choana and nasopharynx, and the turbinates are closely associated with this region [1]. INT specimens have been consistently used as a control in published studies on JNAs [13,20,21]. The cDNA microarray experiments were performed in nine JNA samples and four matched tissues from INT. The expression analysis by quantitative reverse transcription polymerase chain reaction (RT-qPCR) was carried out in 28 samples (including the nine specimens evaluated by cDNA microarray) and eight INT. Protein expression was evaluated in a tissue microarray (TMA) containing 40 formalin-fixed paraffin-embedded (FFPE) samples (including eight JNAs used for cDNA microarray). None of the patients had received radiotherapy or chemotherapy before surgery. Tumor samples were reassessed by three pathologists (S.D.S.W., M.A.C.D. and C.E.F.-A.) to confirm the diagnosis and immunohistochemical findings. Patients were classified according to Radkowski’s staging system [22]. Three patients presented recurrence, and the follow-up was lost in four patients.

The eligibility criteria included JNA patients naïve of chemotherapy or radiotherapy, with no history of a second primary tumor, and submitted to treatment in the same institution. Clinical and pathological data were retrieved from the medical records. The median age of patients was 17.9 years at diagnosis, ranging from 10 to 25 years. This study was approved by the Brazilian Ethics Committee (CONEP 241/2005), and all patients provided written informed consent.

### 2.2. RNA Extraction and Screening for the Identification of DEGs by cDNA Microarray Analysis

Total RNA was isolated using the TRIzol reagent (Life Technologies, Carlsbad, CA, USA), following the manufacturer’s instructions. The RNA quantity and quality were evaluated using NanoDrop^®^ (ND-1000 Spectrophotometer v.3.0.1, Thermo Fisher Scientific, Wilmington, DE, USA) and Bioanalyzer (RNA 6000 NanoLabChip kit/2100 Agilent Technologies, Palo Alto, CA, USA), respectively. The screening was performed using a customized cDNA platform (2.3K platform) containing tumor-related genes, including representative genes of the WNT pathway, PI3K pathway, epithelial–mesenchymal transition (EMT) process, and retinoic acid and neuronal differentiation pathways, among others (Table S5) [23,24].

RNA amplification was performed in two rounds based on T7 polymerase in vitro transcription. Labeled cDNA was generated using six μg of amplified RNAs from the test (tumor and INT specimens) and commercial reference sample (Integrated DNA Technologies, Coralville, IA, USA), random hexamer primers, Cy3- or Cy5-labeled dCTP, and SuperScript II (Thermo Fisher Scientific, Waltham, MA, USA). Hybridization, washing, data acquisition, and normalization were performed as previously described [24].

The normalized cDNA microarray data were uploaded into the TIGR Multiexperiment Viewer (TMeV) software package [25]. Significance analysis of microarray (SAM) was applied to identify differentially expressed genes using 1000 permutations and delta of 2.1 (false positive rate = 1.2) [26].

### 2.3. Gene Expression Analysis by Quantitative Reverse Transcription Polymerase Chain Reaction (RT-qPCR)

We selected eight DEGs to evaluate the expression levels using RT-qPCR, including *BCL2* (BCL2 apoptosis regulator)*, CAV1* (caveolin 1)*, LAMB1* (laminin subunit beta 1)*, CD74* (CD74 molecule), *COL4A2* (collagen type IV alpha 2 chain), *FZD7* (frizzled class receptor 7), *ING1* (inhibitor of growth family member 1), and *RAC2* (Rac family small GTPase 2). A set of these genes was selected based on our previous study of copy number alterations (such as *COL4A2*, *LAMB1*, and *CAV1* mapped in regions involved in gains, and *BCL2* and *CD74* in losses) [13] or its functional role in cancer. The primer pairs were designed (Primer-Blast software, http://www.ncbi.nlm.nih.gov/tools/primer-blast/, accessed on 11 January 2021) to match microarray probes of the selected transcript, which potentially increases the similarity between cDNA microarray and RT-qPCR results (Table S1).

The endogenous controls used for data normalization in the RT-qPCR assays were experimentally chosen. The most stable genes among a panel of six genes were detected using the GeNorm software, as previously described [27]. The reference transcripts tested had an M value (geNorm expression stability coefficient) below 1, indicating a low variability expression among 14 samples (nine tumors and five normal tissues). Since *GAPDH* (glyceraldehyde-3-phosphate dehydrogenase), *GUSB* (glucuronidase beta), and *RPLP0* (ribosomal protein lateral stalk subunit P0) were the most stable transcripts, they were selected as reference genes. The geometric means of these transcripts were used as a single value for reliable normalization of the expression data of the target transcripts. The reactions were performed in duplicates and constructed by robotic pipetting (QIAgility, Qiagen, Courtaboeuf, France) at a total volume of 12.5 μL containing Power SYBR Green PCR Master Mix (Applied Biosystems; Foster City, CA, USA), 20 ng of cDNA, and 200 nM of each primer. The cycling conditions were initially held at 95 °C for 10 min; 40 cycles at 95 °C for 15 s; 58–59 °C for 1 min; followed by a dissociation curve in a 7500 Real time PCR System (Applied Biosystems, Foster City, CA, USA). RT-qPCR experiments were performed under the recommendations of the minimum information for publication of quantitative real-time PCR experiments (MIQE) guideline [28]. Relative gene expression was quantified by the 2^−ΔΔCT^ method [29].

The comparison between cDNA microarray and RT-qPCR results was performed using the SPSS 17.0 (SPSS; Chicago, IL, USA). Dot plot graphs were created with GraphPad Prism 8.0 (GraphPad Software Inc., La Jolla, CA, USA). Nonparametric Mann–Whitney U-test was used to compare normal and tumor groups using a significance level of *p* < 0.05.

### 2.4. Tissue Microarray (TMA) Platform and Immunohistochemistry (IHC)

Core biopsies obtained from defined tumor areas were captured using a Tissue Microarrayer (Beecher Instruments^®^, Silver Spring, MD, USA). The TMA was constructed with 40 JNAs, including eight cases evaluated by the cDNA microarray analysis and one placenta (control sample). Tissue cores with a dimension of 1.0 mm from each specimen were punched and arrayed in duplicate on a recipient paraffin block and spaced 0.2 mm apart. The sections obtained from the recipient block were cut and transferred with adhesive tape to coated slides for subsequent UV cross-linkage (Instrumedics Inc.^®^, Hackensack, NJ, USA). Then, the slides were dipped in a layer of paraffin to prevent oxidation and kept at −20 °C.

We investigated the protein expression of six DEGs, namely BCL2, CAV1, CD74, FZD7, RAF1 (Raf-1 proto-oncogene, serine/threonine kinase), and WNT5A (WNT family member 5A), as well as WNT5B (WNT family member 5B) (details in Table S2). The IHC reactions were performed using a citrate buffer pH 6.0 in a pressure cooker (Pascal^®^, Dako Cytomation, Carpinteria, CA, USA) for antigen retrieval. Then, the unspecified proteins were blocked with skimmed milk at 6% concentration diluted in distilled water for 30 min. The endogenous peroxidase was blocked using a commercial solution (Dako Cytomation, Carpinteria, CA, USA) for 15 min and the primary antibodies were applied. A polymer system was used as a secondary antibody (Envison^®^, Dako Cytomation, Carpinteria, CA, USA) for 60 min, and 3,3′-Diaminobenzidine (Dako Cytomation, Carpinteria, CA, USA) was used as chromogen for 5 min. The tissue samples were counterstained with Harris hematoxylin for 1 min. The isotype protein (negative control) and the primary antibody were used in the same concentration. Positive controls were selected according to the Human Protein Atlas recommendations (https://www.proteinatlas.org/, accessed on 24 June 2021), as follows: a lymphoid tissue for BCL2, a normal nasopharyngeal tissue for FZD7 and WNT5A, and an adenoid sample for CAV1, RAF1, and WNT5B.

We used the ImageJ 1.49v software (National Institutes of Health, MD, USA) to analyze the expression of the proteins [30]. Ten high-power fields from each section were selected, and 100 cells from each field were counted at a final magnification of 400×. Then, the percentage of cells showing antibody reactivity was scored as 0 (<5% of tumor area stained), 1 (5–10% of tumor area stained), 2 (11–50% stained), or 3 (>50% stained). Tumors were also classified by the intensity of staining and scored as 1 (weak), 2 (moderate), or 3 (intense). A final score was calculated by adding intensity and extent scores for each sample, which distinguished four classes: negative (final score 0), low (final score 1–2), moderate (final score 3–4), and strong expression (final score 5–6). The analysis was carried out by two observers (M.A.C.D. and C.E.F.A), and, in case of discrepant recording, a consensus score was used.

### 2.5. In Silico Analyses

Morpheus web-based tool (https://software.broadinstitute.org/morpheus/, accessed on 13 April 2021) was used to generate the heatmap and subsequent analysis of the differential expression results based on cDNA microarray analysis. Euclidean distance and average linkage were used for non-supervised hierarchical clustering.

The ontology and pathway analyses were performed using Enrichr (https://maayanlab.cloud/Enrichr/, accessed on 13 April 2021), an online bioinformatic tool that integrates several gene-set libraries and allowed us to rank enriched terms based on the differentially expressed gene list extracted from the transcriptome analysis [31,32]. The top ten terms in GO, KEGG (*Kyoto Encyclopedia of Genes and Genomes*), NCI-Nature, and BioCarta gene-set libraries were selected according to their adjusted *p*-values.

The web-based eXpression2Kinases (X2K) application (https://maayanlab.cloud/X2K/, accessed on 16 June 2021) was used to identify putative upstream regulators responsible for observed patterns in transcriptome analysis [33]. Transcription factor enrichment analysis (TFEA) and Genes2Networks (G2N) modules of X2K were used to determine the enriched upstream transcription factors (TFs) and the intermediate proteins that connect them in a regulatory network that modulates the expression of the DEGs. Applying the default parameters, the top TFs were ranked based on hypergeometric *p*-value, and the inferred network was generated and visualized.

## 3. Results

### 3.1. Identification of DEGs Involved in Tumor-Related Pathways and Biological Processes, and Their Putative Upstream Regulators

**Figure 1:**
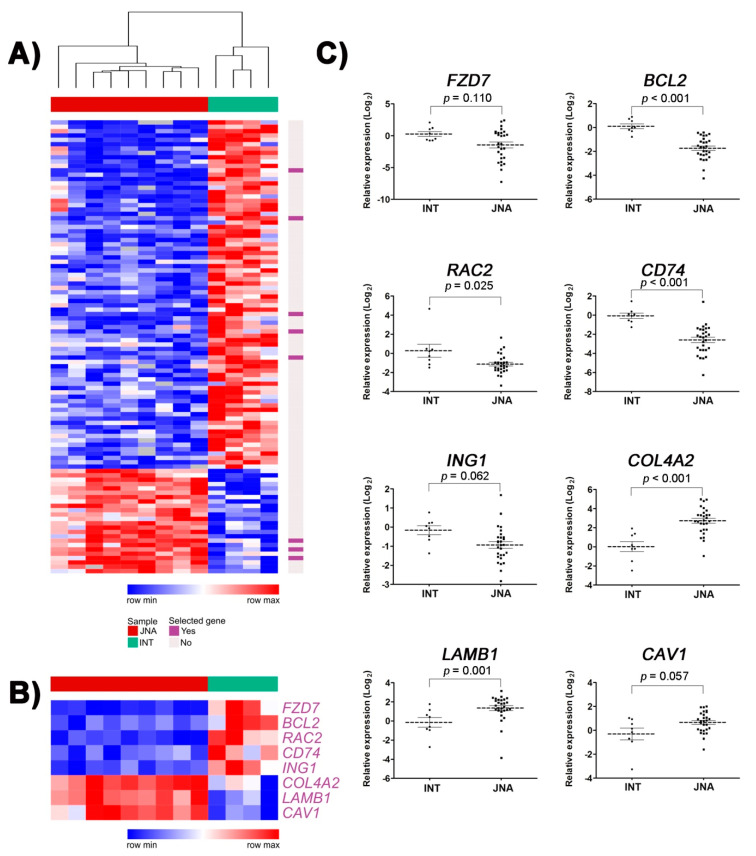
Gene expression analysis in juvenile nasopharyngeal angiofibroma (JNA): (**A**) heatmap and dendogram based on the expression levels of the 104 differentially expressed genes (DEGs) identified by cDNA microarray. Hierarchical clustering was performed assuming Euclidean distance. The top bar identifies JNA (dark red) and inferior nasal turbinate (INT; green) samples, and rows represent genes. Red and blue represent increased and decreased expression, respectively. White indicates no change in expression level compared with the reference sample and gray indicates that no intensity was detected; (**B**) among these DEGs, eight (purple) were evaluated by RT-qPCR and/or immunohistochemistry; (**C**) dot plot showing the normalized relative expression levels from the INT (*n* = 8) and JNA (*n* = 28) samples assessed by RT-qPCR. *p*-value was determined by the Mann–Whitney test.

**Figure 2:**
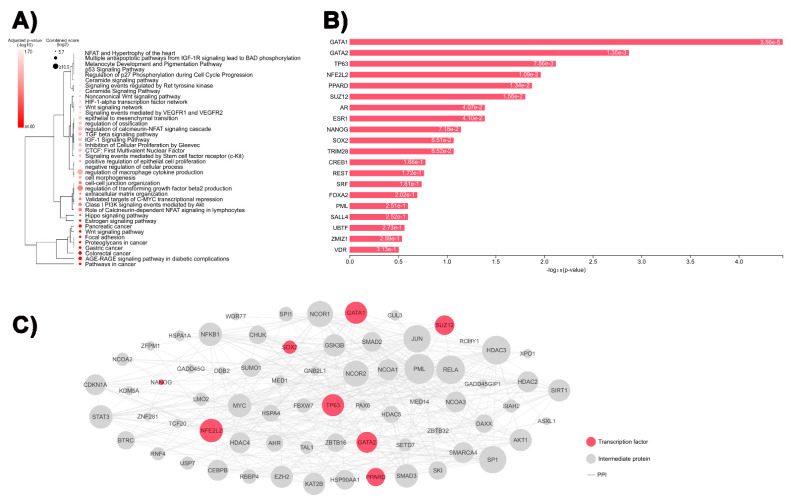
Enriched downstream pathways and upstream transcription factors (TFs) identified by in silico analyses: (**A**) bubble heatmap representing enriched gene ontologies and pathways for the 104 differentially expressed genes (DEGs) from Figure 1; (**B**) top 20 enriched TFs putatively involved in the regulation of the DEGs; (**C**) upstream regulatory network of the DEGs showing protein–protein interactions (PPIs) between the top-ranked TFs and their intermediate proteins. The size of the nodes is proportional to their degree.

We detected 80 down- and 24 upregulated genes when comparing nine JNA with four matched INT samples (Tables S6 and S7). The unsupervised hierarchical clustering analysis showed clear discrimination between JNA and INT (Figure 1A). The 2.3K microarray platform used for large-scale expression analysis included cancer-related genes, such as those involved in the WNT/PI3K pathway and EMT process. Thus, we explored the involvement of the complete list of DEGs in biological processes and pathways. The top 10 biological processes and pathways enriched in JNAs, according to the GO, KEGG, NCI-Nature, and BioCarta gene-set libraries, obtained with the Enrichr tool, are presented in Table S3. As expected, the Enrichr tool analysis underscored the WNT signaling pathway, focal adhesion, colorectal cancer, and pathways in cancer as the most statistically significant JNA-related pathways (Figure 2A). Using the complete list of DEGs as an input, we performed in silico analyses to infer the most likely transcription factors that control this set of genes. Using the X2K online tool, we determined the top 20 enriched TFs putatively involved in regulating the DEGs (Figure 2B) and the upstream regulatory network connecting the top-ranked TFs and their intermediate proteins (Figure 2C).

### 3.2. Validation of Selected DEGs by RT-qPCR

To confirm the expression pattern detected in the microarray analysis, eight DEGs were selected and evaluated by RT-qPCR: five presented decreased expression (*FZD7*, *BCL2*, *RAC2*, *CD74*, and *ING1*), and three genes showed increased expression levels (*COL4A2*, *LAMB1*, and *CAV1*) (Figure 1B). In the technical validation (nine JNAs and four INT specimens), all transcripts presented concordance between cDNA microarray and RT-qPCR results (Table S4). The same expression tendency was observed for all tested transcripts in an independent set of 19 JNAs and four normal samples (biological validation). *BCL2*, *COL4A2*, and *LAMB1* genes sustained their significant differential expression on tumors compared to the normal samples (Table S4). When all samples were combined, *COL4A2* and *LAMB1* showed a significantly increased expression (*p* ≤ 0.001), whereas *BCL2* (*p* < 0.001), *CD74* (*p* < 0.001), and *RAC2* genes (*p* = 0.025) presented a significantly decreased expression in JNAs compared to the normal samples (Figure 1C). *CAV1* overexpression (*p* = 0.057) and *ING1* down expression (*p* = 0.062) showed a trend towards statistical significance.

### 3.3. Immunohistochemistry Analyses

**Figure 3:**
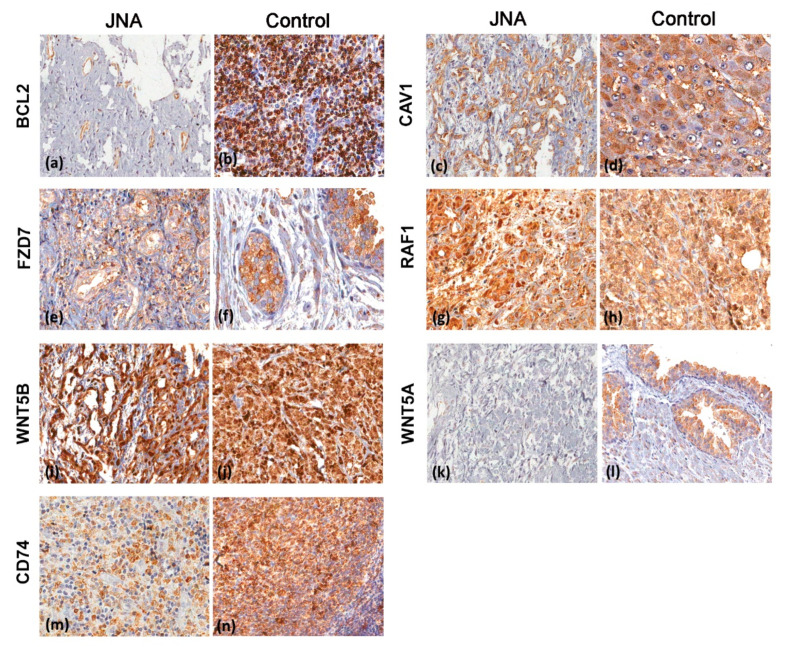
Immunohistochemical analysis of different markers in 40 juvenile nasopharyngeal angiofibroma (JNA) and control samples: (**a**) moderate cytoplasmic BCL2 immunoexpression in the vascular structures of a JNA sample; (**b**) strong cytoplasmic BCL2 expression in a positive control (lymphoid tissue); (**c**) strong diffuse cytoplasmic and membranous CAV1 in neoplastic cells and absent expression in stromal cells; (**d**) strong cytoplasmic CAV1 expression in an adenoid sample; (**e**) moderate FZD7 expression in vascular structures and stromal cells of a JNA sample; (**f**) a normal nasopharyngeal sample (control) positive for FZD7; (**g**) strong cytoplasmic RAF1 expression in neoplastic, inflammatory, and stromal cells in JNA sample; (**h**) strong RAF1 expression in a positive control sample; (**i**) strong cytoplasmic WNT5B expression in vascular structures and inflammatory and stromal cells of a JNA sample; (**j**) strong WNT5B expression in the positive control; (**k**) negative WNT5A expression in neoplastic and stromal cells in a JNA sample; (**l**) WNT5A in a positive control tissue; (**m**) diffuse moderate membranous CD74 staining in neoplastic and inflammatory cells of a JNA sample and its positive control (**n**) Harris’ hematoxylin counterstaining.

TMA was performed to investigate whether changes in the gene expression pattern in JNAs could affect the protein expression. Seven proteins were evaluated in a TMA containing 40 JNAs (including eight cases evaluated in the cDNA microarray) and one placenta sample as a control (Figure 3, Table 1). Moderate to strong granular cytoplasmic expression was observed for CAV1 in the vascular structures of JNAs. BCL2 immunoreactivity was low in endothelial and stromal JNA components. Negative to moderate FZD7 expression was observed in endothelial cells, whereas RAF1 presented a strong cytoplasmic expression in endothelial, inflammatory, and stromal cells. The CD74 showed moderate to strong membranous expression in endothelial and inflammatory cells. WNT5A was absent or low in endothelial and stromal components. In contrast, the WNT5B expression was strong in the endothelial and stromal cells of the JNA samples. The protein expression profiles of BCL2, CAV1, CD74, FZD7, RAF1, WNT5A, and WNT5B in the JNA specimens were compiled in Table 1.

## 4. Discussion

To date, the molecular events that drive JNA development remain to be established, and rare studies have been undertaken to overcome the lack of effective biomarkers and therapeutic targets. The present study provides the gene expression profile of JNAs using a customized platform containing crucial cell signaling pathways for tumor growth and progression. Gene expression patterns showed a clear difference between normal and tumor tissues, revealing 104 DEGs.

One of these DEGs, *BCL2*, showed significant downregulation in JNAs compared to INT samples. In agreement with the transcript-level results, negative to low expression of the BCL2 protein was found in most cases evaluated in the TMA. In JNAs, Pauli et al. (2008) reported focal staining of BCL2 in small vessel endothelial cells; however, the absence of BCL2 immunostaining was observed in stromal cells [34]. Similarly, a set of our JNA specimens exhibited moderate BCL2 immunostaining in the vascular structures. Some studies suggested that the absence of BCL2 was correlated with poorly differentiated tumors (breast carcinomas) or associated with an unfavorable prognosis (soft tissue tumors) [35,36]. In other cases, such as desmoids tumors and nodular fasciitis, BCL2 reactivity was also consistently negative in tumor cells, with BCL2 reactivity only in scattered small lymphocytes [37]. BCL2 is an anti-apoptotic protein involved in cell proliferation, survival, and differentiation, particularly in neuronal differentiation [38].

Two DEGs, *RAC2* (downregulated) and *RAF1* (upregulated), were associated with the major biological processes and pathways implicated in JNA development. RAC2 is a member of the Ras superfamily of small guanosine triphosphate (GTP)-metabolizing proteins, and regulates diverse cellular events, including the control of cell growth, neutrophil motility, and cytoskeletal reorganization [39,40]. Although *RAC2* is primarily expressed by hematopoietic stem and progenitor cells [41], recent reports have implicated *RAC2* overexpression in the development and progression of malignant tumors of the brain, kidney, and lung [42,43,44]. We confirmed RAF1 upregulation at the protein level in JNA samples, exhibiting strong staining in endothelial, inflammatory, and stromal cells. *RAF1* was previously implicated in the carcinogenic process in human cancers and associated with tumor angiogenesis [45]. The differential expression of *RAC2* and *RAF1* genes was validated in the microarray-independent set of JNA samples at transcriptional and protein levels. Since these genes are recurrently involved in the enriched KEGG pathways detected after the bioinformatics analysis, it is reasonable to hypothesize that they are key regulators of JNA development.

CD74 is a transmembrane protein that stabilizes the major histocompatibility complex (MHC) type II in the endoplasmic reticulum, enabling the presentation of MHCII-restricted antigens at the cell surface. Therefore, CD74 is crucial for the activation of adaptive immunity against tumor cells [46]. Here, gene expression analyses using macro-dissected tumor tissues (cDNA microarray and RT-qPCR) revealed consistent *CD74* decreased expression, whereas the IHC analysis showed moderate to strong immunopositivity in endothelial and inflammatory cells in most JNA samples. Considering the heterogeneous composition of the tumor microenvironment, a fundamental limitation in bulk-cell analyses, such as RT-qPCR, is that they are unable to resolve differences in the gene expression deriving from the differential infiltration of immune and other cell populations rather than from differences in tumor cells [47]. In addition, DEGs associated with immune and inflammatory functions are responsive to the percentage of tumor cells in macro-dissected tissues [48]. This explains the apparent discordant results when comparing the transcriptional expression levels of *CD74* with the immunoreactivity restricted to the endothelial and inflammatory cells observed in the tumoral tissue. Importantly, CD74 protein overexpression was detected in some malignant tumors, including cervical squamous cell carcinoma, urothelial bladder carcinoma, and in inflammatory bowel disease [49,50,51]. Zhang et al., 2021 reported that CD74 upregulation promotes the invasive ability of pancreatic ductal adenocarcinoma cells and modulates the expression of *GDNF* (glial cell-derived neurotrophic factor) via the AKT/EGR-1 pathway, enhancing perineural invasion [52].

From the eight selected DEGs, only *COL4A2* and *LAMB1* exhibited a significant upregulation. Alterations in the extracellular matrix (ECM) components modify the tumor microenvironment and the crosstalk between cancer cells and nonmalignant cells that surround the tumor. Consequently, these alterations can modulate tumor growth, angiogenesis, and metastasis. Collagen IV and laminin are structural components of the basal membrane and play a key role in anchoring the single-layered epithelium [53]. The association of collagen IV, laminin, and matrix metallopeptidase 9 expression was demonstrated in colorectal cancer cells, in which there was an abnormal accumulation of laminin in the cytoplasm with an absence of basal membranes containing collagen IV [54,55,56]. Recent studies showed a significantly higher expression of ECM components, including collagen IV and laminin, in colorectal liver metastases and central nervous system metastasis, particularly in desmoplasia areas [57,58]. These findings illustrate how the tumor-associated stroma contribute to shaping the tumor behavior.

An additional oncoprotein involved in ECM organization, apoptosis, cell migration, and metastasis is CAV1 [59,60]. Although we found a trend to the enhanced expression of the *CAV1* gene in the combined microarray-dependent and -independent validation sets, the moderate to strong protein expression was detected in 29 of 37 JNAs evaluated by IHC. Therefore, CAV1 might also have an influence on JNA behavior.

The WNT signaling pathway is implicated in developmental processes and tissue homeostasis, acting on the maintenance of stem-like properties and the control of cell proliferation, migration, and survival under physiological conditions [61]. The WNT signaling is usually divided into three pathways: canonical β-catenin/T cell factor, planar cell polarity, and the Ca^2+^ pathway. This intricate network comprises 19 glycolipoprotein ligands, including WNT5A. We confirmed the downregulation of WNT5A at transcriptional and protein levels in JNA specimens. Previous studies reported that 45% to 75% of breast cancer patients presented negative or lower expression of the WNT5A protein, which has been associated with disease progression and metastasis, and a poor recurrence-free survival [62]. Additionally, we investigated another ligand involved in the WNT pathway, WNT5B, which exhibited a strong immunopositivity in JNAs. Harada et al., 2017 demonstrated that exosomes secreted WNT5B, which acts in a paracrine fashion to promote cancer cell migration and proliferation [63]. WNT5B overexpression was previously detected in several tumor types (such as non-small cell lung cancer, chronic lymphocytic leukemia, and basal-like breast cancer) and was associated with cancer aggressiveness and a poorer prognosis [64,65]. In head and neck squamous cell carcinomas, increased WNT5B expression was shown to promote the invasive ability of tumor-derived cell lines through the upregulation of matrix metallopeptidase 10 [66,67]. Recent clinical trials are currently in development using WNT inhibitors, such as monoclonal antibodies [68]. For instance, Vantictumab (OMP_18R5, clinical trials NCT02005315, NCT01973309, NCT01345201, and NCT01957007) directly binds to frizzled (FZD) receptors and blocks the binding of WNT ligands to FZD, including FZD7. We found moderate to high FZD7 expression in 59% of our cases. Therefore, patients showing an increased expression of this regulator could benefit from this targeted therapy.

In addition to our major focus, which was to build upon the current knowledge of the JNAs gene expression profile, we used online tools to identify pathways and upstream regulators related to the DEGs. Although our customized microarray platform included a selected subset of genes and was centered on cancer-related pathways, these in silico analyses enabled us to detect the preferential distribution of the DEGs among the pathways integrating the platform. Thus, the main overrepresented pathways were pancreatic cancer, the WNT signaling pathway, focal adhesion, proteoglycans in cancer, gastric cancer, colorectal cancer, the AGE-RAGE signaling pathway in diabetic complications, and pathways in cancer. Based on the DEGs, our analyses also revealed TFs, such as SOX2 (SRY-box transcription factor 2) and NANOG (Nanog homeobox), that were putatively involved in the upstream regulation of these genes. These TFs are fundamental for the maintenance of the cancer stem cells’ (CSCs) self-renewal ability [69]. Developmental signaling pathways, including the WNT and Hippo pathways, are often altered in CSCs [70]. The regulatory functions exerted by such pathways support the retention of stem-like properties and the development of treatment resistance [71].

Besides the WNT pathway effects on cancer cells, evidence consistently suggests that the canonical and non-canonical aberrant signaling in the tumor microenvironment promote EMT, metastasis, and CSC maintenance [72]. Therefore, the WNT pathway activity might integrate JNA cells with the tumor microenvironment, establishing crosstalk between the main pathways and putative upstream regulators explored in our study. A set of studies and clinical trials in human cancers has investigated targeted drugs and biological agents that could hopefully be repurposed to treat JNAs. For instance, the treatment of BLBC cell lines with LGK-974, which inhibits the secretion of WNT proteins, achieved promising results that support its applicability for WNT5B targeted therapy [64]. A WNT5A-mimicking molecule, formylated WNT5A-derived hexapeptide or Foxy5, has been tested to reestablish the WNT5A signaling in human breast epithelial cells, and its potential as an anti-metastatic agent has been demonstrated through pre-clinical and clinical phase I studies [62,73]. Treatment based on WNT signaling regulators could be an efficient therapy for JNA patients.

## 5. Conclusions

We described gene expression alterations followed by the validation of the main DEGs involved in cell proliferation, ECM structure, and stemness maintenance. Altogether, our study revealed potential biomarkers that can contribute to JNA pathogenesis. Further investigation might provide new avenues for targeted therapy for these benign and locally aggressive tumors.

## Figures and Tables

**Table 1:** Protein expression results obtained in juvenile nasopharyngeal angiofibromas evaluated by immunohistochemistry.

	Protein—Number of Cases (%)						
Final Score	BCL2	CAV1	CD74	FZD7	RAF1	WNT5A	WNT5B
Negative	14 (35.9)	5 (13.5)	2 (5.3)	13 (38.2)	0	29 (78.4)	1 (2.7)
Low	18 (46.2)	3 (8.1)	3 (7.9)	1 (2.9)	0	3 (8.1)	0
Moderate	7 (17.9)	18 (48.7)	23 (60.5)	13 (38.2)	1 (2.6)	5 (13.5)	7 (18.9)
Strong	0	11 (29.7)	10 (26.3)	7 (20.7)	38 (97.4)	0	29 (78.4)
Total	39	37	38	34	39	37	37

## Data Availability

The cDNA microarray data presented in this study are available in the Supplementary Tables S5–S7.

## Supplementary Materials

The following are available online at https://www.mdpi.com/article/10.3390/biomedicines9091258/s1: Table S1, primer sequences for the reference genes and selected transcripts evaluated by RT-qPCR; Table S2, antibodies used to investigate the protein expression by immunohistochemistry; Table S3, relevant biological processes and pathways associated with the differentially expressed genes according to the Enrichr tool analysis. The top 10 enriched terms in the BioCarta, Gene Ontology (GO), Kyoto Encyclopedia of Genes and Genomes (KEGG), and NCI-Nature gene-set libraries are listed.; Table S4, comparison between tumor and normal samples for each transcript and analysis category; Tables S5–S7, customized microarray platform and dataset used to identify differentially expressed genes in juvenile nasopharyngeal angiofibromas.
