# Supplementary material for: Master Regulators of Epithelial-Mesenchymal Transition and WNT Signaling Pathways in Juvenile Nasopharyngeal Angiofibromas
Source: Biomedicines. 2021 Sep 18;9(9):1258. doi: 10.3390/biomedicines9091258 (PMC8469518; doi:10.3390/biomedicines9091258)
Supplement: Supplementary file 1 [file biomedicines-09-01258-s001.zip › biomedicines-1346828-SI.pdf]

Table S1. Primer sequences for the reference genes and selected transcripts evaluated by RT-qPCR.

Gene symbol	Primer sequences	Amplicon length
	**Reference Genes**	
*GAPDH**	F - 5'TGCACCACCAACTGCTTAG3'<br>R - 5'GATGCAGGGATGATGTTC3'	176bp
*GUSB**	F - 5'GAAAATACGTGGTTGGAGAGCTCATT3'<br>R - 5'CCGAGTGAAGATCCCCTTTTTA3'	101bp
*RPLP0**	F - 5'GGAGACGCATTACACCTTC3'<br>R - 5'CTTCACCTTAGCTGGGG3'	139bp
	**Candidate Genes**	
BCL2	F - 5'TGTGTGGAGAGCGTCAACCG3'<br>R - 5'TCAGAGACAGCCAGGAGAAATCA3'	181bp
CAV1	F - 5'ACCTCCTCACAGTTTTCATCCAGC3'<br>R - 5'GCCTTGTTGTTGGGCTTGTAGAT3'	127bp
CD74	F - 5'GCTTTTCCATCCTGGTGACTCTGC3'<br>R - 5'AGGCTTGGGAGGCTTGGGA3'	155bp
COL4A2	F - 5'GGGGTGGAAAGGTGACGCT3'<br>R - 5'CCCGTTGATGCCTGCGAA3'	106bp
FZD7	F - 5'TGGAGTTCTTTGAAATGTGCTGGAA3'<br>R - 5'GCTCCCATGATTCTCTGCTAAGCT3'	125bp
ING1	F - 5'GAGTCCCTGCCTTTCGACTTGC3'<br>R - 5'GCGCTCGTAGCACTCGTCTA3'	102bp
LAMB1	F - 5'AAGCTGCCCAAAACTCCGGG3'<br>R - 5'CGGCTTTCCTTCTGGCATCAGC3'	177bp
RAC2	F - 5'CACCGACACTCTCCAGGCTC3'<br>R - 5'GTCCACCATCACATTGGCTGAA3'	169bp

F = forward primer; R = reverse primer; * reference genes

Table S2. Antibodies used to investigate the protein expression by immunohistochemistry.

Antibody	Catalog number	Clone	Host	Dilution	Manufacturer
Anti-BCL2	IS61430-2	124	Mouse monoclonal	1:300	Dako-Agilent, Santa Clara, CA, USA
Anti-CAV1	ab32577	E249	Rabbit monoclonal	1:250	Abcam, Cambridge, UK
Anti-CD74	sc-6262	LN-2	Mouse Monoclonal	1:100	Santa Cruz Biotechnology, Dallas, TX, USA
Anti-FZD7	GWB-E6553E	-	Rabbit Polyclonal	1:200	Genway Biotech, San Diego, CA, USA
Anti-RAF1	ab181115	EP4969	Rabbit monoclonal	1:250	Abcam, Cambridge, UK
Anti-WNT5A	ab235966	-	Rabbit Polyclonal	1:300	Abcam, Cambridge, UK
Anti-WNT5B	ab150602	-	Rabbit Polyclonal	1:400	Abcam, Cambridge, UK

(-) polyclonal antibodies.

Table S3. Relevant biological processes and pathways associated with the differentially expressed genes according to the Enrichr tool analysis. The top 10 enriched terms in the BioCarta, Gene Ontology (GO), Kyoto Encyclopedia of Genes and Genomes (KEGG), and NCI-Nature gene-set libraries are listed.

Enriched terms	Adjusted *p*-value ($-\log_{10}$)	Combined Score ($\log_2$)	Genes
**BioCarta**			
TGF beta signaling pathway	2.263694899	8.280064325	*TGFB2; SMAD4; CDH1*
IGF-1 Signaling Pathway	2.263694899	8.280064325	*MAPK8; IGF1; RAF1*
Inhibition of Cellular Proliferation by Gleevec	2.263694899	8.070895405	*MAPK8; BCL2; RAF1*
CTCF: First Multivalent Nuclear Factor	2.263694899	7.973733049	*TGFB2; SMAD4; CDKN1B*
Ceramide Signaling Pathway	1.888179013	7.188755253	*MAPK8; BCL2; RAF1*
NFAT and Hypertrophy of the heart	1.704595592	6.560751421	*MAPK8; EDN1; IGF1*
Multiple antiapoptotic pathways from IGF-1R signaling lead to BAD phosphorylation	1.704595592	7.800489752	*IGF1; RAF1*
Melanocyte Development and Pigmentation Pathway	1.704595592	7.800489752	*BCL2; RAF1*
p53 Signaling Pathway	1.704595592	7.800489752	*BCL2; TIMP3*
Regulation of p27 Phosphorylation during Cell Cycle Progression	1.704595592	7.800489752	*CDKN1B; SKP1*
**GO**			
Cell-cell junction organization	2.897650209	7.935079405	*CDH6; TJP1; TGFB2; CDH3; CDH1; CLDN1*
Regulation of transforming growth factor beta2 production	2.897650209	11.32001038	*SMAD4; TGFB2; CDH3*
Extracellular matrix organization	2.897650209	6.772564798	*MMP11; POSTN; TGFB2; COL4A2; CDH1; SERPINE1; ICAM3; LAMB1; LOXL2*
Regulation of macrophage cytokine production	2.45462084	10.15038089	*CD74; TGFB2; WNT5A*
Cell morphogenesis	2.45462084	7.718758321	*CDH6; TGFB2; CDH3; CDH1; RAC2*
Positive regulation of epithelial cell proliferation	2.430283943	7.041373946	*CDH3; FZD7; WNT5A; LAMB1; SOX9; IGF1*
Negative regulation of cellular process	2.430283943	5.729957251	*AZGP1; TGFB2; SMAD4; CDKN1B; FTH1; CAV1; PDCD5; BHLHE40; BCL2; PPT1; RAF1; ING1*
Epithelial to mesenchymal transition	2.226162771	7.949843228	*TGFB2; WNT5A; SOX9; LOXL2*
Regulation of ossification	2.226162771	7.891397721	*TGFB2; WNT5A; SOX9; PRKACA*
Regulation of calcineurin-NFAT signaling cascade	2.226162771	9.080132971	*RCAN1; RCAN3; IGF1*
**KEGG**			
Pathways in cancer	6.466298191	7.236709876	*TGFB2; SMAD4; EDN1; CDKN1B; FZD7; WNT5A; LAMB1; IGF1; MAPK8; COL4A2; CDH1; BCL2; RAC2; RAC3; PRKACA; RAF1; SKP1*
AGE-RAGE signaling pathway in diabetic complications	5.323196874	8.256314229	*TGFB2; SMAD4; EDN1; MAPK8; CDKN1B; COL4A2; SERPINE1; BCL2*
Colorectal cancer	4.695419164	8.101592401	*MAPK8; TGFB2; SMAD4; RAC2; BCL2; RAC3; RAF1*

Focal adhesion	4.463028935	7.111825763	*MAPK8; COL4A2; CAV1; BCL2; RAC2; RAC3; LAMB1; IGF1; RAF1*
Proteoglycans in cancer	4.463028935	7.08791045	*TGFB2; FZD7; CAV1; WNT5A; ITPR1; TIMP3; IGF1; PRKACA; RAF1*
Gastric cancer	4.463028935	7.345942753	*TGFB2; SMAD4; CDKN1B; CDH1; FZD7; WNT5A; BCL2; RAF1*
Wnt signaling pathway	4.337917928	7.20898761	*SMAD4; MAPK8; FZD7; WNT5A; RAC2; RAC3; PRKACA; SKP1*
Pancreatic cancer	4.213871002	7.84405439	*MAPK8; TGFB2; SMAD4; RAC2; RAC3; RAF1*
Estrogen signaling pathway	3.808795298	7.040826917	*KRT19; KRT15; ITPR1; BCL2; KRT13; PRKACA; RAF1*
Hippo signaling pathway	3.414787704	6.675556184	*TGFB2; SMAD4; CDH1; YWHAB; FZD7; SERPINE1; WNT5A*
**NCI-NATURE**			
Role of Calcineurin-dependent NFAT signaling in lymphocytes	3.000308833	7.955865881	*RCAN1; MAPK8; YWHAB; BCL2; PRKACA*
Validated targets of C-MYC transcriptional repression	2.930105281	7.573292518	*SMAD4; CDKN1B; FTH1; WNT5A; BCL2*
Class I PI3K signaling events mediated by Akt	2.930105281	8.135257715	*CDKN1B; YWHAB; PRKACA; RAF1*
Signaling events mediated by Stem cell factor receptor (c-Kit)	2.319674044	7.202381679	*MAPK8; GRB10; BCL2; RAF1*
HIF-1-alpha transcription factor network	2.111786644	6.672375961	*EDN1; SMAD4; SERPINE1; BHLHE40*
Wnt signaling network	2.111786644	7.545603799	*IGFBP4; FZD7; WNT5A*
Signaling events mediated by VEGFR1 and VEGFR2	2.111786644	6.605374227	*CAV1; GRB10; PRKACA; RAF1*
Noncanonical Wnt signaling pathway	2.021480507	7.255617428	*MAPK8; FZD7; WNT5A*
Signaling events regulated by Ret tyrosine kinase	1.851701712	6.881629075	*MAPK8; GRB10; PRKACA*
Ceramide signaling pathway	1.777919174	6.560751421	*MAPK8; BCL2; RAF1*

Table S4. Comparison between tumor and normal samples for each transcript and analysis category.

Genes	cDNA microarray		Array dependent validation		Array independent validation	
	Fold[#] (T/N)	P	Fold[#] (T/N)	P	Fold[#] (T/N)	P
BCL2	-6.29	0.005*	-1.47	0.064	-5.10	0.002*
CAV1	6.71	0.005*	2.44	0.045*	1.19	0.516
CD74	-2.56	0.005*	-5.49	0.045*	-8.64	0.006*
COL4A2	5.53	0.005*	16.62	0.045*	3.68	0.023*
FZD7	-10.56	0.005*	-1.70	0.165	-3.62	0.292
ING1	-2.74	0.005*	-1.98	0.123	-2.09	0.019*
LAMB1	4.83	0.005*	2.14	0.045*	4.17	0.029*
RAC2	-4.05	0.005*	-3.42	0.045*	-1.78	0.168

* Mann-Whitney statistical test significance ($P<0.05$); [#]Fold based on median of tumor (T) and normal samples (N).
